# Girdin Promotes Tumorigenesis and Chemoresistance in Lung Adenocarcinoma by Interacting with PKM2

**DOI:** 10.3390/cancers14225688

**Published:** 2022-11-19

**Authors:** Fuyang Cao, Desong Yang, Feiyu Tang, Can Lu, Xiang He, Songming Chen, Zhanghuan Yang, Siyuan Gong, Lunquan Sun, Atsushi Enomoto, Masahide Takahashi, Liang Weng

**Affiliations:** 1 Department of Oncology, Xiangya Cancer Center, Xiangya Hospital, Central South University, Changsha 410008, China; 2 Key Laboratory of Molecular Radiation Oncology Hunan Province, Xiangya Hospital, Central South University, Changsha 410008, China; 3 Hunan Clinical Medical Research Center of Accurate Diagnosis and Treatment for Esophageal Carcinoma, Hunan Cancer Hospital and The Affiliated Cancer Hospital of Xiangya School of Medicine, Central South University, Changsha 410013, China; 4 Thoracic Surgery Department 2, Hunan Cancer Hospital and The Affiliated Cancer Hospital of Xiangya School of Medicine, Central South University, Changsha 410013, China; 5 Department of Pathology, Xiangya Hospital, Central South University, Changsha 410008, China; 6 Hunan International Science and Technology Collaboration Base of Precision Medicine for Cancer, Changsha 410008, China; 7 Institute of Gerontological Cancer Research, National Clinical Research Center for Gerontology, Changsha 410008, China; 8 Center for Molecular Imaging of Central South University, Xiangya Hospital, Central South University, Changsha 410008, China; 9 Department of Pathology, Nagoya University Graduate School of Medicine, Nagoya 466-8550, Japan; 10 International Center for Cell and Gene Therapy, Fujita Health University, Toyoake 470-1192, Japan; 11 Clinical Research Center for Respiratory Diseases in Hunan Province, Changsha 410008, China; 12 Xiangya Lung Cancer Center, Xiangya Hospital, Central South University, Changsha 410008, China

**Keywords:** Girdin, PKM2, tumorigenesis, chemoresistance, LUAD

## Abstract

**Simple Summary:**

Aerobic glycolysis is a key driving force of tumorigenesis and chemoresistance. Girdin plays a vital role in cancer cells; however, the role of Girdin in aerobic glycolysis is still unclear. In this study, we first found that knockout of Girdin markedly inhibited lung adenocarcinoma (LUAD) progression in an autochthonous LUAD mouse model. In addition, we found that Girdin interacted with pyruvate kinase M2 (PKM2) and impaired PKM2 activity, which promoted the Warburg effect and chemoresistance. Our results suggest that Girdin is a potential therapeutic target to overcome the resistance of LUAD cells to chemotherapeutic agents.

**Abstract:**

Girdin, an Akt substrate, has been reported to promote tumorigenesis in various tumors. However, the role of Girdin in a spontaneous tumor model has not yet been explored. Here, we studied the role of Girdin in lung adenocarcinoma (LUAD) using the autochthonous mouse model and found that Girdin led to LUAD progression and chemoresistance by enhancing the Warburg effect. Mechanistically, Girdin interacted with pyruvate kinase M2 (PKM2), which played a vital role in aerobic glycolysis. Furthermore, Girdin impaired Platelet Derived Growth Factor Receptor Beta (PDGFRβ) degradation, which in turn, promoted PKM2 tyrosine residue 105 (Y105) phosphorylation and inhibited PKM2 activity, subsequently promoting aerobic glycolysis in cancer cells. Taken together, our study demonstrates that Girdin is a crucial regulator of tumor growth and may be a potential therapeutic target for overcoming the resistance of LUAD cells to chemotherapy.

## 1. Introduction

Lung cancer is the second most malignant tumor type in terms of incidence, occupying the top spot for mortality worldwide [1,2]. Non-small cell lung cancer (NSCLC) is the most common pathological type of lung cancer, accounting for approximately 85% of all cases, while lung adenocarcinoma (LUAD) is the most common type of NSCLC. Remarkable advances have been made in recent years in the treatment of lung cancer, including surgery, radiotherapy, chemotherapy, targeted therapy, and immunotherapy. However, patients frequently do not receive the maximum benefit from chemotherapy due to their late-stage diagnosis and high metastasis of LUAD, and chemoresistance developed by tumors severely limits the effectiveness of treatment and greatly impairs the survival of patients. Therefore, classifying the biological functions and drug resistance mechanism of LUAD must be addressed in order to provide patients with more effective therapy.

Tumor cells convert large amounts of glucose to lactate even in the presence of oxygen in a process known as the Warburg effect or aerobic glycolysis [3]. The Warburg effect promotes cell proliferation, migration, and drug resistance [4]. Pyruvate kinase (PK) is the key enzyme that catalyzes the final step of glycolysis, converting phosphoenolpyruvate (PEP) to pyruvate while phosphorylating ADP to ATP [5]. PK is encoded by the *PKM*-gene and then spliced into Pyruvate kinase M1 and M2 (PKM1 and PKM2). PKM1 has high activity and is expressed in almost all tissues. In contrast, PKM2 is mainly expressed in rapidly proliferative cells, such as normal embryonic and tumor cells. PKM2 promotes the synthesis of macromolecules such as nucleic and amino acids, which are indispensable for cell proliferation. It has been shown that the activity of PKM2 is regulated by its phosphorylation [6]. The receptor tyrosine kinase (RTK) FGFR disrupts the formation of active tetrameric PKM2 by directly phosphorylating PKM2 Y105 and releasing the cofactor fructose-1,6-bisphosphate [6,7]. In tumor cells, PKM2 acts as a molecular switch that determines whether glucose is converted to lactate for energy regeneration (active tetrameric form, the Warburg effect) or used for cellular anabolism (almost inactive dimeric form) [8], reprogramming tumor metabolism and regulating cell growth, metastasis, and chemoresistance. However, the regulation of PKM2 activity by phosphorylation is not fully understood; therefore, it is vital to further explore the specific regulatory mechanisms of PKM2 in LUAD to improve the prognosis of patients.

Girdin is an actin-binding protein that is highly expressed in breast cancer [9], glioblastoma [10], and colorectal cancer [11]. Girdin interacts with Akt (Serine/Threonine Kinase 1) [12,13], the trimeric Gαi/s [14], and dynamin [15], participating in multiple biological processes such as proliferation [16], migration [9,12], angiogenesis [12,17], endocytosis [18], and apoptosis [19]. Although several studies implicate Girdin in the promotion of tumorigenesis [9,10,11,20], these studies used a transplanted tumor model in mice, which cannot fully reflect tumor initiation and progression in vivo. Therefore, exploring the role of Girdin in a spontaneous tumorigenesis animal model is necessary to dissect Girdin’s effect on tumor formation. In addition, multiple biological processes regulated by Girdin are closely related to energy metabolism. For example, Girdin has been shown to regulate the mechanistic target of the rapamycin (mTOR) signaling pathway [21] to promote cell growth and produce chemoresistance by modulating autophagy to reprogram energy metabolism in pancreatic cancer cells [22]. However, whether Girdin is involved in tumor metabolism regulation remains unclear.

In this study, we showed for the first time the role of Girdin in tumorigenesis with an inducible autochthonous tumorigenesis model of LUAD. We also showed that Girdin interacts with PKM2 and inhibits PKM2 activity, thereby shifting glycolysis from mitochondrial respiration to lactate production. Our data indicate that Girdin is greatly indispensable for the proliferation, aerobic glycolysis, and chemoresistance of tumor cells.

## 2. Materials and Methods

### 2.1. Generation of Lenti-sgRNA/Cre Virus

#### 2.1.1. sgRNA Construction

Lenti-sgNT/Cre vectors encoding individual sgRNAs were purchased (#66895, Addgene, Watertown, MA, USA). Fragments containing sg*Trp53* or sg*Trp53*-sgGirdin were synthesized by GENEWIZ (Suzhou, China) with the cloning sites of PmeI and AscI. They were then introduced in tandem at the site of the original U6-sgRNA cassette. The primer sequences used for cloning sgRNAs and assembling multi-sgRNA vectors are listed in Table 1.

#### 2.1.2. Lentivirus Package

Lenti-sgRNA/Cre virus was produced by co-transfection with packaging vectors psPAX2 (#12260, Addgene, Watertown, MA, USA) and envelope plasmid pCMV-VSV-G (#8454, Addgene, Watertown, MA, USA) into 293FT cells using Lipo293™ (C0521, Beyotime, Shanghai, China). Viral supernatants were collected 48 h after post-transfection and then passed through a 0.45 μm filter (SLHP033RB, Millipore, Darmstadt, Germany), concentrated with EZ Lentivirus concentrated solution (AC04L442, Life-iLab, Shanghai, China), and resuspended in sterilized PBS (E607008-0500, Sangon Biotech, Shanghai, China). Lentivirus was divided and stored at −80 °C.

#### 2.1.3. RT-PCR Measurements of Lentivirus Titer

The concentrated lentivirus was used to infect HEK293 cells in 5 μL, 10 μL, and 20 μL for 24 h, and the medium was changed for fresh DMEM (Gibco, Carlsbad, CA, USA) containing 10% FBS (Excell, Shanghai, China) to harvest for 48 h. The total DNA was extracted using Ezup Column Animal Genomic DNA Purification kits (B518251-0100, Sangon Biotech, Shanghai, China). Real-time PCR amplifications were performed as follows: 95 °C for 30 s, 95 °C for 10 s, 55 °C for 0:15 + Plate Read, 68 °C for 30 s, (GO TO) 95 °C for 10 s (39 more times), Melt Curve 65 to 95 °C for increment 0.5 °C for 0:05 + Plate Read. The copy number of the lentivirus was calculated based on the standard curve depicted, using pLenti-sgNT/Cre and pRK5-HA-GST-RagB as the standards [23]. The primer sequences used for Lentivirus titer are listed in Table 2. WPRE is an element of Lenti-sgRNA/Cre plasmid and RagB is the reference gene.

### 2.2. Ethics Statement, Mice, and Tumor Initiation

Ethics statement: All animal studies were approved by the Institutional Animal Care and Use Committee (IACUC) of Central South University. All in vivo operations were performed in compliance with Central South University’s Animal Facility regulations.

Mice: *Kras^LSL-G12D/+^* mice were purchased from Shanghai Biomodel Organism Science & Technology Development Co., Ltd. (NO. NM-KI-190003, Shanghai, China); *Rosa26^LSL-Cas9-tdTomato^* mice were purchased from Gempharmatech Co., Ltd. (NO. T002249, Nanjing, China). All animals were maintained on a mixed C57BL/6J 3 129SvJ genetic background. The two genetic mice were crossed to obtain *KCT* mice (*Kras^LSL-G12D/+^*; *Rosa26^LSL-Cas9-tdTomato^*). All the experimental mice were genotyped at least three times; primers are listed in Table 3.

Tumor initiation: After being anesthetized with avertin, mice between 6 and 8 weeks of age were treated via nasal inhalation of lentivirus carrying sgRNAs and Cre recombinase (5 × 10^5^ IFU/mouse), as described earlier [23,24,25,26].

### 2.3. Histology and Immunohistochemistry

Mice were sacrificed and their lungs were dissected and fixed with 10% formalin neutral buffer overnight at room temperature. Fixed tissues were embedded in paraffin, sectioned at 5 mm, and then hematoxylin and eosin (H&E) stained (Department of Pathology, Xiangya Hospital, Central South University, Changsha, China). The total lung area occupied by the tumor was measured on H&E-stained slides using K-Viewer software. Immunohistochemistry was performed as described [21] and analysis was conducted using Mantra 1.0.3 and inform 2.4.0. The antibodies used were: Girdin (A16132, ABclonal, Wuhan, China), Ki-67 (A16667, abcam, Cambridge, UK), and TTF1(PA352, abcarta, Suzhou, China).

### 2.4. Measurement of Glucose Assumption, Lactate, and ATP Production

Glucose, lactate, and ATP concentrations of the cultured medium were measured by using glucose (GO) assay (GAGO20-1KT, Sigma-Aldrich, St. Louis, MO, USA), L-Lactate assay (MAK329-1KT, Sigma-Aldrich, St. Louis, MO, USA) and ATP assay (MAK190-1KT, Sigma-Aldrich, St. Louis, MO, USA) kits according to the manufacturer’s instructions. Glucose consumption, lactation, and ATP production were normalized by amounts of cellular protein. The data are means SD from three independent experiments.

### 2.5. PKM2 Activity Assay

A549 and KP Cells were washed twice with ice-cold PBS, lysed with a PK assay buffer (K709, BioVision, Waltham, MA, USA), and then collected. PKM2 activity was determined using a PK activity assay kit (K709, BioVision, Waltham, MA, USA) according to the manufacturer’s instructions. The activity of PKM2 was corrected by the number of cells. The data means SD from three independent experiments.

### 2.6. Cell Culture, Transfection, Plasmids, and RNA Interference

A549 cells were purchased from the American Type Culture Collection (Rockville, MD, USA), the human embryonic kidney epithelial 293FT cell line was purchased from Invitrogen (Carlsbad, CA, USA), and the KP cells derived from an autochthonous tumorigenesis mouse model (Cre induced LUAD in *Kras^LSL-G12D/+^*; *Trp53^f/f^* mice) were kindly provided by Prof. Hongbin Ji (Shanghai Institutes for Biological Sciences, Chinese Academy of Sciences). A549 cells were cultured in RPMI-1640 (Gibco, Carlsbad, CA, USA) supplemented with 10% fetal bovine serum (FBS, Excell, Shanghai, China). KP and 293FT cell lines were cultured in DMEM (Gibco, Carlsbad, CA, USA) supplemented with 10% FBS. Plasmids and siRNA were transfected into cells using Lipo8000^TM^ (C0533, Beyotime, Shanghai, China) and LipoRNAi^TM^ (C0535, Beyotime, Shanghai, China) according to the manufacturer’s instructions.

The N-terminal fragment of Girdin (NT) was cloned and inserted into the pGEX-6P-3 vector (#27-4599-01, Addgene, Watertown, MA, USA). GFP, GFP-NT, GFP-M1, GFP-M2, and GFP-CT cDNAs were inserted into the pRetroQ-3xFlag vector as previously described [22]. sgRNAs were designed using the CRISPR tool (http://crispr.mit.edu (accessed on 16 September 2019)) to minimize potential off-target effects. The genomic primers and sgRNA sequences are listed in Table 4. The specificity of the siRNA used in this study has been previously identified [12,18,21]; its primers are listed in Table 4.

### 2.7. Antibodies and Reagents

The following antibodies were used for western blot and immunofluorescent studies: Girdin (AF5345, R&D Systems, Minneapolis, MN, USA); Girdin (A16132, ABclonal, Wuhan, China); PKM2 (#4053, Cell Signaling Technology, Danvers, MA, USA); p-PKM2 (#3827, Cell Signaling Technology, Danvers, MA, USA); p53 (#2524, Cell Signaling Technology, Danvers, MA, USA); GST (sc459, Santa Cruz, Santa Cruz, CA, USA); GFP (598, MBL, Tokyo, Japan); β-Actin (66009-1-Ig, Proteintech, Wuhan, China). The uncropped Western Blot figures for relevant results are shown in Figures S2–S5.

### 2.8. Data Analysis

All statistical analyses were conducted using GraphPad Prism 8.0.2 software (GraphPad, San Diego, CA, USA). Data are shown as the means ± SD. Statistical significance was analyzed using Student’s *t*-test. A 2 × 2 Fisher’s Exact Test was used to analyze the number of tumor-bearing mice. Pearson’s chi-squared was conducted for the clinicopathological characteristics of LUAD patients. *p* < 0.05 was regarded as statistically significant.

## 3. Results

### 3.1. Girdin Knockout Severely Inhibits LUAD Tumorigenesis

**Figure 1:**
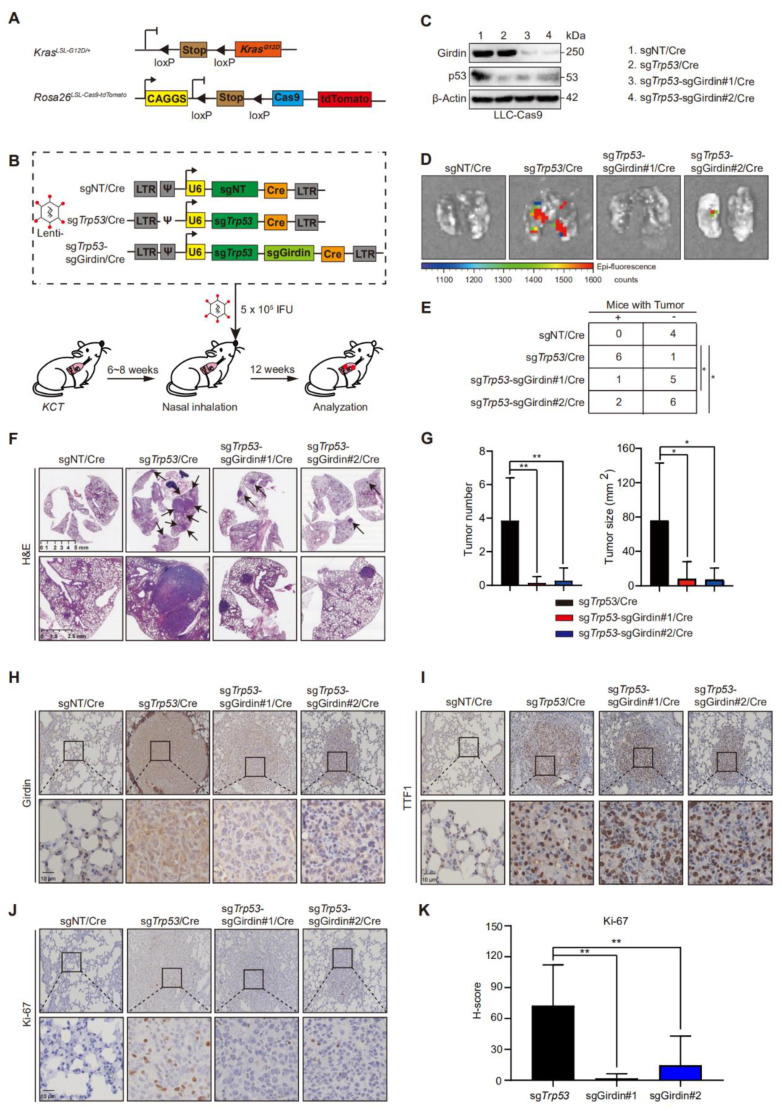
Girdin knockout significantly suppresses autochthonous tumorigenesis in LUAD. (**A**) Schematic of the *Kras^LSL-G12D/+^* allele and *Rosa26^LSL-Cas9-tdTomato^* double knockin in a genetically engineered mouse model (GEMM). (**B**) Lung tumors were initiated in the *KCT* mice and allowed to develop for 12 weeks after intranasal administration of Lenti-sgNT/Cre or Lenti-sgRNAs/Cre. (**C**) Western blot (WB) evaluation of the knockdown efficiency of p53 and Girdin sgRNAs in LLC-Cas9 cells. (**D**) Representative living images of tumor-bearing lungs from *KCT* mice induced (by inhalation) with diverse lentivirus. Excitation maximum: 554 nm; emission maximum: 581 nm. (**E**) Contingency tables demonstrating the number of tumor-bearing mice. (Two-sided Fisher’s exact test, * *p* < 0.05). (**F**) Representative hematoxylin and eosin (H&E) staining of serial sections from lung tumor mice 12 weeks post-intranasal infection (scale bars 5 mm and 1.25 mm). (**G**) Quantification of tumor number (**left**) and size (**right**) per mouse for *KCT* mice 12 weeks post-Lenti-sgNT/Cre or Lenti-sgRNAs/Cre treatment. Data are shown as mean ± SEM. (*T*-test, * *p* < 0.05, ** *p* < 0.01). (**H**–**J**) Representative immunohistochemistry (IHC) for Girdin, TTF1, and Ki-67 in *KCT* mice with sgNT/Cre, sgp53/Cre, or sgp53-sgGirdin/Cre lung adenocarcinoma primary tumors. Scale bar, 10 μm. (**K**) Quantification of Ki-67 staining for (**J**). Data are shown as mean ± SEM. (*T*-test, ** *p* < 0.01).

To evaluate the biological functions of Girdin in vivo, we obtained *Kras^LSL-G12D/+^*; *Rosa26^LSL-Cas9-tdTomato^* (*KCT*) mice by crossing *Kras^LSL-G12D/+^* and *Rosa26^LSL-Cas9-tdTomato^* mice strains (Figure 1A). The mice were genotyped by a classical PCR method (Figure S1A). The tumor was initiated through infection with lentivirus containing a sgRNA sequence targeting the *Trp*53 gene and a Cre recombinase (Lenti-sg*Trp*53/Cre) via nasal inhalation. A non-targeting gRNA (sgNT) was used as a negative control (Lenti-sgNT/Cre). The effect of Girdin on tumorigenesis was evaluated by designing a lentivirus vector containing sgRNA sequences targeting both *Trp*53 and Girdin (Lenti-sg*Trp*53-sgGirdin/Cre) (Figure 1B). The knockout (KO) efficiency was first assessed by infecting LLC cells stably expressing Cas9 with lentivirus (Figure 1C). The efficiency of Girdin KO was further tested by T7 Endonuclease I (T7EI) mismatch assays. The gene editing efficiency of sgGirdin#1 and #2 were 51.2% and 55.4%, respectively (Figure S1B). Furthermore, the DNA sequencing data revealed the induction of several mutations in the Girdin gene locus, including the terminal mutation that shifted TAG to TAA, which terminated gene transcription earlier than usual (Figure S1C), supporting the identification of Girdin depletion at the DNA level.

Twelve weeks after tumor initiation, *KCT* mice transduced with Lenti-sgNT/Cre showed no tumors, while most mice transduced with Lenti-sg*Trp*53/Cre developed many large tumors. Interestingly, the mice infected with Lenti-sg*Trp*53-sgGirdin/Cre showed fewer and smaller tumors, as detected by fluorescence imaging (Figure 1D). The number of tumor-bearing mice was counted. There were no tumor-bearing mice in the negative control group sgNT/Cre. A total of 6 out of 7 (86%) mice in the sg*Trp53*/Cre group developed tumors, while the tumorigenic rates of mice in the sg*Trp53*-sgGirdin group were only 1/6 and 2/8 (Figure 1E). Quantification by histology confirmed that *KCT* mice transduced with Lenti-sg*Trp*53-sgGirdin/Cre had fewer and smaller tumors compared with the positive control (Figure 1F,G). Immunostaining with Girdin antibodies confirmed the successful Girdin KO in the tumors from mice transduced with Lenti-sg*Trp*53-sgGirdin/Cre (Figure 1H), and all the tumors were lung adenocarcinomas, based on positive TTF1 staining (Figure 1I). Girdin KO tumors showed a significant reduction in Ki67 staining, indicating the inhibition of tumor proliferation (Figure 1J,K). Taken together, these data demonstrate that Girdin depletion inhibited autochthonous tumorigenesis of LUAD.

### 3.2. Girdin Promotes LUAD Progression

**Figure 2:**
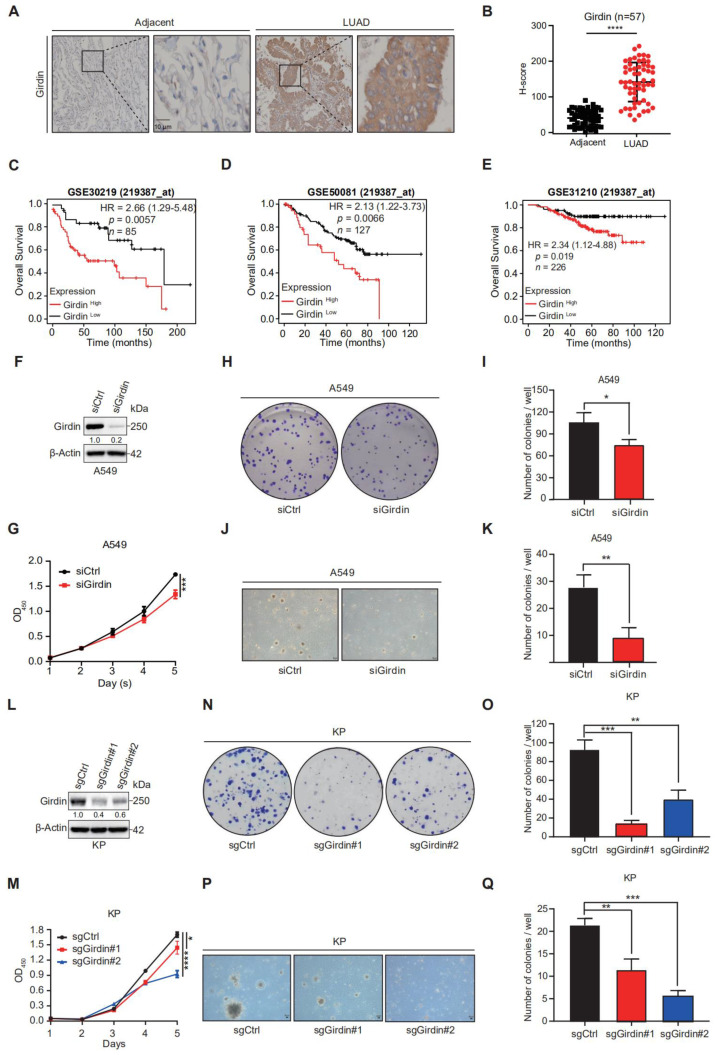
Girdin ablation inhibits cell proliferation in LUAD. (**A**,**B**) IHC staining and statistical analysis of Girdin in human LUAD tissue. Scale bar, 10 μm. *n* = 57. Data are shown as mean ± SEM (**** *p* < 0.0001). (**C**–**E**) Kaplan–Meier analysis of overall survival was performed based on Girdin expression. GSE 30,219, GSE 50,081, and GSE 31,210 are from the Gene Expression Omnibus (GEO) database. (**F**) A549 cells were transfected with siCtrl or siGirdin for 72 h and immunoblotted as shown. (**G**) Effects of siCtrl and siGirdin on cell proliferation were determined by using CCK8 assays with the A549 stable cell line. Data are shown as mean ± SEM (*** *p* < 0.001). (**H**,**I**) Representative images and statistical results of siCtrl and siGirdin for colony formation in the A549 cell line. Data are shown as mean ± SEM (* *p* < 0.05). (**J**,**K**) Representative images and statistical results of siCtrl and siGirdin for soft agar assay in the A549 cell line. Data are shown as mean ± SEM (** *p* < 0.01). (**L**) Efficiency of CRISPR/Cas-mediated knockdown of Girdin in KP cells was shown by WB analyses. (**M**) Effects of sgCtrl and sgGirdin on cell proliferation were determined by CCK8 assays in the KP stable cell line. Data are shown as mean ± SEM (* *p* < 0.05, **** *p* < 0.0001). (**N**,**O**) Representative images and statistical results of sgCtrl and sgGirdin for clone formation in the KP stable cell line. Data are shown as mean ± SEM (** *p* < 0.01, *** *p* < 0.001). (**P**,**Q**) Representative images and statistical results of sgCtrl and sgGirdin for soft agar assay in the KP stable cell line. Data are shown as mean ± SEM (** *p* < 0.01, *** *p* < 0.001).

To further investigate the involvement of Girdin in lung adenocarcinoma, we checked Girdin expression in lung adenocarcinoma tissues by immunohistochemistry. Compared to the paired adjacent normal lungs, higher Girdin expression was detected in cancer tissues (Figure 2A,B). To extend those data, we checked whether Girdin expression was correlated with clinicopathological characteristics for LUAD patients and found that high expression of Girdin suggested an advanced clinical stage (Table 5). Furthermore, we analyzed Girdin’s expression in several datasets; high Girdin expression was associated with worse overall survival (Figure 2C–E). These data indicate that Girdin may promote LUAD progression.

Regarding the role of Girdin in the LUAD, siRNA-mediated Girdin depletion significantly decreased cell proliferation and colony formation of A549 cells in 2D and 3D cultures in vitro (Figure 2F–K). When we knocked out Girdin in KP cells (a LUAD cell line derived from an autochthonous tumorigenesis mouse model) and repeated the above assays, the same results were consistently attained, as in the A549 cells (Figure 2L–Q). Collectively, these results indicate that Girdin ablation can substantially inhibit cell growth in LUAD.

### 3.3. Girdin Enhances the Warburg Effect in LUAD

**Figure 3:**
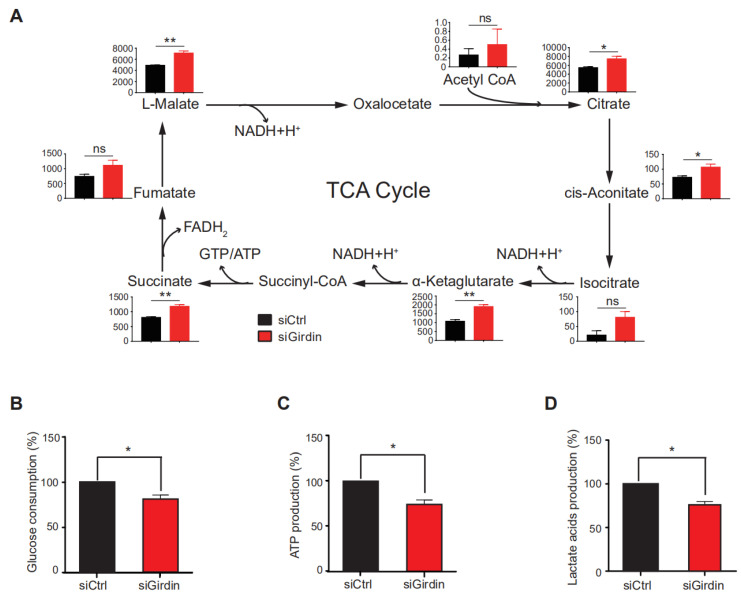
Girdin participates in TCA cycle and glycolysis regulation. (**A**) Metabolomic analysis of cells incubated for 24 h after transfection with indicated siRNAs for 48 h and then labeled with D-(13C6) glucose. The amounts of the indicated metabolites of the TCA cycle were measured (ns, not significant, * *p* < 0.05, ** *p* < 0.01). (**B**) Glucose consumption in A549 cells transfected with siRNAs (* *p* < 0.05). (**C**) ATP production in A549 cells transfected with siRNAs (* *p* < 0.05). (**D**) Lactate acid production in A549 cells transfected with siRNAs (* *p* < 0.05).

Previous studies have shown that Girdin is involved in the regulation of the Akt and mTORC1 signaling pathways [18,21], and these two pathways are closely related to tumor cell metabolism, especially the glycolytic process [27]. To clarify the underlying mechanism by which Girdin mediated the inhibition of lung cancer cell growth, we performed metabolomics analysis, which showed that Girdin was involved in the regulation of the tricarboxylic acid (TCA) cycle. Analysis showed that the intermediates of the TCA cycle, such as citrate, cis-aconitate, α-ketoglutarate, succinate, and L-malate, were significantly upregulated in Girdin-interfering (siRNA) A549 cells (Figure 3A). In addition, we examined glucose consumption, ATP production, and lactate production in Girdin-depleted A549 cells. We found that Girdin knockdown inhibited glucose consumption (Figure 3B) as well as ATP and lactate production (Figure 3C,D). Altogether, these results suggest that Girdin can promote the Warburg effect by shifting the TCA cycle to the process of lactate production, which may contribute to its role in lung cancer cell growth.

### 3.4. Identification of PKM2 as a Girdin-Interacting Protein

**Figure 4:**
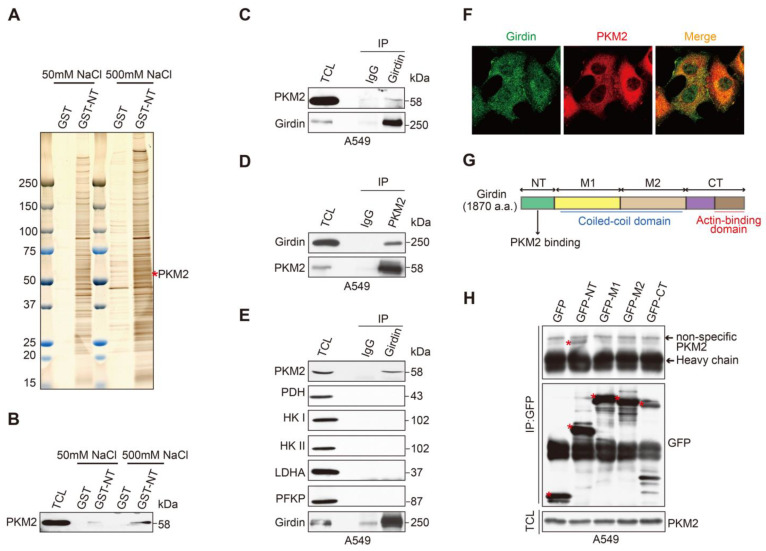
Identification of PKM2 as a Girdin-interacting protein. (**A**) Girdin-interacting proteins were isolated by purification from *E. coli* and visualized with silver staining. (**B**) Direct interaction between Girdin and PKM2 by GST pull-down assay. (**C**,**D**) Endogenous IP using control IgG and anti-Girdin or anti-Flag antibody was carried out in A549 cells, showing that Girdin interacts with PKM2 in the LUAD cell line. (**E**) Co-immunoprecipitation (co-IP) was performed in A549 cells to screen the Girdin-interacting proteins from key enzymes of glycolysis. (**F**) Representative confocal images of Scheme 2 in A549 cells. Girdin was detected using an anti-Girdin primary antibody and Alexa Fluor 488 goat anti-mouse antibody, and PKM2 was detected using an anti-PKM2 primary antibody and Alexa Fluor 594 goat anti-rabbit antibody. (**G**) Domain structure of human Girdin protein. The N-terminal (NT) domain is responsible for the interaction with PKM2. (**H**) PKM2 interacts with Girdin by binding to its NT domain. Lysates from 293FT cells transfected with the Girdin fragments fused with GFP were immunoprecipitated with the anti-GFP antibody, followed by WB analyses using the PKM2 antibody. Bound PKM2 is indicated by a red asterisk.

To explore the mechanism of Girdin involved in the regulation of glycolysis, we employed affinity column chromatography to isolate Girdin interacting proteins. To this end, we purified the glutathione S-transferase (GST)-tagged Girdin amino-terminal domain (GST-NT) from *Escherichia coli*, since GST-NT showed stable and high expression levels in this bacterial expression system. Glutathione sepharose beads coated with purified GST protein or GST-NT were incubated with whole-cell lysate from A549 cells. The interacting proteins eluted by high-salt solutions were analyzed by mass spectrometry, which identified pyruvate kinase M2 (PKM2) as one of the Girdin-interacting proteins (Figure 4A). Due to the critical role of PKM2 in the Warburg effect, we speculated that Girdin may regulate lung cancer aerobic glycolysis through interaction with PKM2.

The interaction between PKM2 and the Girdin NT domain suggested by affinity column chromatography was confirmed by western blot analysis (Figure 4B). The Girdin-PKM2 interaction was further confirmed by reciprocal co-IP in A549 cells (Figure 4C,D). In addition, we performed the co-IP to detect the interaction between Girdin and several enzymes in the glycolysis pathway. Again, only PKM2 and no other enzymes such as pyruvate dehydrogenase (PDH), hexokinase (HK), Lactate Dehydrogenase A (LDHA), or Phosphofructokinase Platelet (PFKP) interacted with Girdin (Figure 4E). Furthermore, immunofluorescence (IF) staining was conducted with Girdin or PKM2 antibodies in A549 cells to detect their subcellular localization. The results showed that Girdin and PKM2 were mainly co-localized in the cytoplasm (Figure 4F). Mapping the interacting domains indicated that the Girdin NT domain was responsible for the association with PKM2 (Figure 4G,H). All the above results demonstrate that PKM2 is a bonafide Girdin-interacting protein in lung cancer cells.

### 3.5. Girdin Leads to Chemoresistance by Regulating PKM2 Phosphorylation and Activity

**Figure 5:**
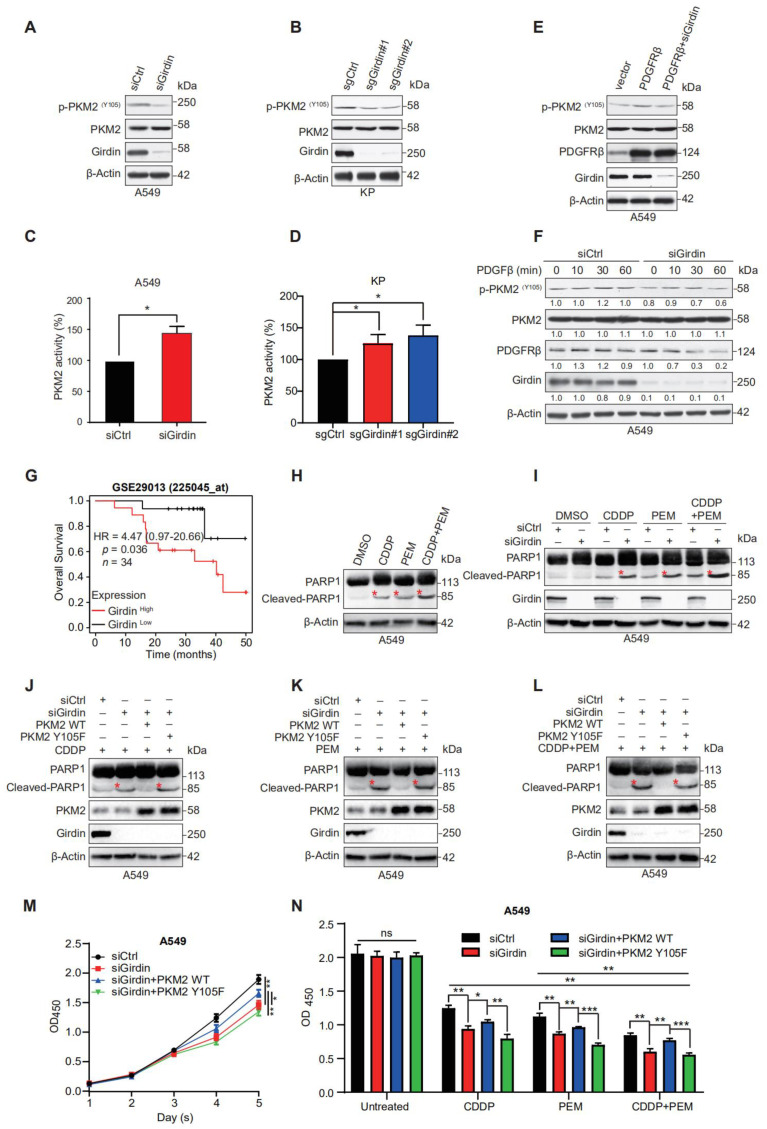
Girdin develops chemoresistance via PKM2. (**A**,**B**) The phosphorylation levels of PKM2 were assessed by WB using anti-p-PKM2 (Tyr 105) antibodies. Cell extracts from A549 cells transfected with siRNAs for 72 h (**A**) and the KP-Girdin KO stable cell line (**B**). (**C**,**D**) PKM2 kinase activity was detected in A549 and KP cell lines with siRNA or sgRNAs (* *p* < 0.05). (**E**) The phosphorylation levels of PKM2 were assessed after vector or PDGFRβ plasmids or PDGFRβ plus siGirdin was transfected for 48 h in the A549 WT cell line. (**F**) Control or Girdin-interfering A549 cells were treated with PDGβ for the indicated time points, followed by WB analysis of p-PKM2, p-PDGFRβ, Girdin, and PKM2 with cell lysates. (**G**) Overall survival (OS) after chemotherapy in NSCLC patients with low and high expression of Girdin; data from the Kaplan-Meier plotter. (**H**) A549 WT cells were treated with CDDP (10 μM), PEM (2 μM), and “CDDP + PEM” for 48 h; WB analysis of indicated proteins in the cell lysates was performed. (**I**) A549 cells were transfected with siRNAs for 24 h and then treated with CDDP (10 μM), PEM (2 μM), or “CDDP + PEM” for 48 h. WB analysis of indicated proteins in the cell lysates was performed. (**J**–**L**) A549 cells stably expressing PKM2 WT or the PKM2 Y105F mutant were transfected with siRNAs for 24 h and then treated with CDDP (10 μM), PEM (2 μM), or “CDDP + PEM” for 48 h. WB analysis of indicated proteins in the cell lysates was performed. The red asterisks in (**H**–**L**) indicated the cleaved bands of PARP1 after inducing with corresponding chemotherapeutics. (**M**) CCK8 assay was performed in A549 cells transfected with siCtrl, siGirdin, siGirdin+ PKM2 WT, or siGirdin + PKM2 Y105F (* *p* < 0.05, ** *p* < 0.01). (**N**) Cell survival assay was conducted in cells transfected the same as (**M**), but cells were treated with CDDP (10 μM), PEM (2 μM), or “CDDP + PEM” for 48 h (ns, not significant, * *p* < 0.05, ** *p* < 0.01, *** *p* < 0.001).

PKM2 is a key enzyme involved in the Warburg effect and provides a growth advantage to tumors. Phosphorylation of PKM2 at Y105 promotes the Warburg effect and tumor growth, while Giridn ablation inhibits the proliferation in LUAD cells. We next investigated the effect of Girdin on phosphorylated Y105 of PKM2. The results showed that Girdin depletion attenuated the phosphorylation of PKM2 in A549 and KP cells (Figure 5A,B). Previous studies reported that the phosphorylation level of PKM2 was negatively correlated with its activity [6,28], so we continued to detect the effect of Girdin on the activity of PKM2. Consistently, Girdin knockdown significantly increased PKM2 activity (Figure 5C,D). These results suggest that Girdin promotes the Warburg effect by regulating PKM2 phosphorylation and activity.

We next explored how Girdin regulates PKM2 phosphorylation. As tyrosine is mainly phosphorylated by tyrosine kinase, we tested the effect of several tyrosine kinases on PKM2 phosphorylation. We found that platelet-derived growth factor receptor β (PDGFRβ) could promote PKM2 Y105 phosphorylation as enhanced phosphorylation of PKM2 was detected in A549 cells overexpressing PDGFRβ. However, Girdin knockdown markedly reduced p-PKM2 expression induced by PDGFRβ, suggesting that Girdin was not dispensable in cells overexpressing PDGFRβ (Figure 5E). Interestingly, Girdin depletion increased PDGβ-induced degradation of PDGFRβ and thus inhibited PDGF signaling and PKM2 phosphorylation in lung cancer cells (Figure 5F). These results suggest that Girdin can reduce PKM2 activity by inhibiting PDGFRβ degradation.

Given that aerobic glycolysis is associated with drug resistance in cancer cells [29], we examined whether Girdin promotes resistance of cancer cells to chemotherapeutic agents. Cisplatin (CDDP), a cell cycle non-specific cytotoxic drug targeting DNA, is a broad-spectrum chemotherapy drug for lung cancer. Pemetrexed (PEM), a kind of first-line drug for LUAD, especially for progressive LUAD, is an anti-folate agent with a core of pyrrolopyrimidine moieties in its structure that inhibits tumor growth by disrupting the normal intracellular folate-dependent metabolic process and suppressing cell replication. To achieve the best chemotherapy outcome, a “CDDP + PEM” regimen is always regarded as the first-line treatment strategy for patients diagnosed with late-stage LUAD. To investigate the role of Girdin on the chemotherapeutic effect of LUAD, we found that the LUAD patients with high Girdin expression showed poor prognosis after receiving chemotherapy according to the Kaplan-Meier plotter database (Figure 5G).

To further investigate the effect of Girdin on chemotherapy treatment for LUAD, we treated A549 cells with CDDP, PEM, and “CDDP + PEM” and detected the apoptosis marker cleaved-PARP1 at first. CDDP and PEM indeed induced cell apoptosis, and the two pharmaceuticals had a synergistic effect on LUAD cells by inducing cell apoptosis (Figure 5H). When cells were transfected with siCtrl or siGidin, similar operations were conducted again, and it was clear that Girdin interference promoted cell apoptosis when cells were given different chemotherapeutic drugs. Furthermore, we noticed a dramatic promotion of cell apoptosis in Girdin depletion cells following combinational use of CDDP and PEM compared with using them separately, which indicated that Girdin ablation could increase chemotherapy sensitivity (Figure 5I). Moreover, in Girdin-interfering cells, increased apoptosis was restored by forced expression of PKM2 WT but not the PKM2 Y105F mutant, no matter what drug was administered (Figure 5J–L). Taken together, our results suggest that Girdin promotes chemoresistance in the first-line treatment for LUAD.

To determine the role of PKM2 and Girdin in promoting chemoresistance to CDDP and PEM in cell survival, we checked the cell proliferation in Girdin-depleted A549 cells with overexpression of PKM2 WT or Y105F mutant. Unexpectedly, PKM2 WT reverted the effect on proliferation caused by siGirdin, but PKM2 Y105F failed to exert a similar influence (Figure 5M), indicating that Girdin-mediated PKM2 Y105 phosphorylation augments chemoresistance. Then, we performed a survival assay by administering CDDP, PEM, or “CDDP + PEM” to cells transfected with Girdin siRNA and PKM2 rescued. Girdin-depletion promoted CDDP- and PEM-induced cell apoptosis, which can be rescued by forced expression of PKM2 WT but not its Y105F mutant. Otherwise stated, Girdin facilitated chemoresistance by promoting PKM2 phosphorylation, and consistent synergies could be observed when cells were given “CDDP + PEM” (Figure 5N). Collectively, these results show that Girdin leads to chemoresistance by promoting cell proliferation and PKM2 Y105 phosphorylation in lung cancer cells.

## 4. Discussion

**Figure 6:**
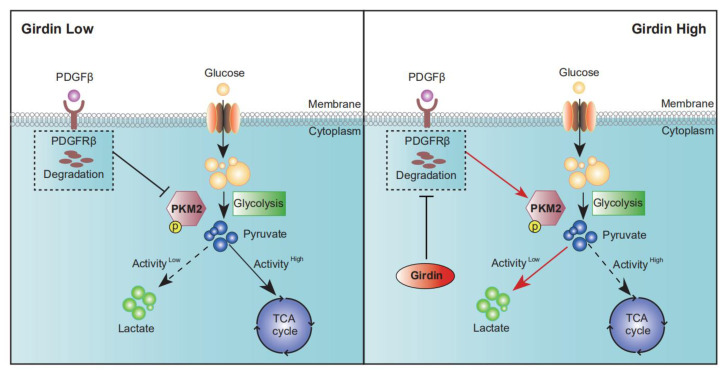
Girdin enhances the Warburg effect by restraining PDGFRβ degradation and PKM2 activity. A hypothetical model illustrating the functional role of Girdin in the Warburg effect. Girdin inhibits PKM2 activity by blocking PDGFRβ degradation, relieving its suppression effect on PKM2 phosphorylation, and eventually up-regulating the Warburg effect.

In this study, we revealed that Girdin is involved in the regulation of aerobic glycolysis in LUAD cells through the inhibition of PKM2 activity, leading to cancer cell growth and chemoresistance. This process may be related to Girdin-mediated inhibition of PDGFRβ degradation (Figure 6).

Girdin has been found to play an important role in promoting tumor cell proliferation [30], metastasis [11], angiogenesis [17], and signal transduction [21]. Girdin acts as an Akt substrate and is involved in the activation of the PI3K/Akt signaling pathway. Akt enhances the Warburg effect of tumor cells by increasing the activity of factors such as glucose transporters (GLUT), hexokinase (HK), and phosphofructokinase 1 (PFK1). Akt also promotes protein and lipid synthesis by activating mTORC1. At the molecular level, these metabolic changes likewise activate hypoxia-inducible factor 1 (HIF-1), which drives cellular energy metabolism to lactate production. However, all of the studies related to Girdin utilized transplanted tumor mouse models [9,22], which cannot reflect the real status of tumorigenesis in vivo.

It was reported that the *Kras^G12D^* mutant coordinated with *Trp53* deficiency could drive spontaneous LUAD tumor formation in a mouse model, which has proven to be an excellent model to mimic LUAD tumorigenesis in vivo. In our study, we showed for the first time that Girdin is a critical factor governing the development of LUAD by using this model and identified the mechanism of Girdin on the Warburg effect in LUAD cells, extending our understanding of the importance of Girdin in tumor development.

The activity of PKM2 is regulated by its phosphorylation, and the reported phosphorylation sites of PKM2 are Y83, Y105, Y148, Y175, Y370, and Y390. Hitosugi et al. (2009) found that fibroblast growth factor receptor type 1 (FGFR1) disrupted PKM2 activity by directly phosphorylating PKM2 Y105. FGFR1 disrupted the binding of PKM2 to the co-factor fructose-1,6-bisphosphate, thereby inhibiting the formation of active tetrameric PKM2 [6]. Min Tian et al. (2021) found that Anexelekto (AXL) inhibited the activity of PKM2 and promoted aerobic glycolysis by phosphorylating PKM2, which was relevant to cisplatin-mediated drug resistance in ovarian cancer [31]. In this study, we found that Girdin regulated the phosphorylation of PKM2, possibly by a mechanism that involves inhibiting platelet-derived growth factor receptor (PDGFRβ) degradation, which may be regulated by the intracellular transport function of Girdin, as previously reported [21].

Of course, many other RTKs play an important role in the development of LUAD, and these must be explored in the future. In terms of chemoresistance, the Girdin-mediated Warburg effect may not be the only mechanism affecting chemoresistance, but it plays a crucial role in LUAD treatment. Since PKM2 Y105 phosphorylation can be induced by several tyrosine kinases (FGFR1, ITK, PTK2/FAK, YES, AXL, CLK1, EphA4, TNK2/ACK1, JAK3, and Src) [6,32] and PKM2 activity can be regulated by cell cycle-related mechanism, how Girdin regulates PKM2 phosphorylation may not be through cell cycles but by another mechanism, and this requires further study. Future studies should be conducted to uncover how Girdin regulates PKM2 via direct interaction with PKM2.

In conclusion, our study reveals that Girdin promotes tumorigenesis in vitro and in vivo; further analysis finds that Girdin interacts with PKM2 and inhibits PKM2 activity, causing chemoresistance in LUAD. Girdin increases metabolic flux to glycolysis and concomitantly inhibits entry into mitochondrial respiration in cancer cells. TEPP-46 and Mitapivat, two inhibitors reported to target PKM2 in glucose metabolism [33], should be tested to investigate their roles in overcoming CDDP and PEM resistance in LUAD patients with up-regulated Girdin expression.

## 5. Conclusions

In summary, we showed that Girdin promoted LUAD growth both in vivo and in vitro and demonstrated that Girdin promotes the Warburg effect by modulating PKM2 activity which, in turn, leads to tumorigenesis and chemoresistance. Our results suggest that Girdin may be a potential therapeutic target to overcome the resistance of LUAD cells to chemotherapeutic agents.

## Figures and Tables

**Table 1:** The sequence of primers for Lenti-sgRNA/Cre plasmids.

Primer	Sequences (5′ to 3′)
Lenti-sg*Trp53*/Cre-F	AGCTTTGTTTAAACGAGGGCCTATTTCCCATGAT
Lenti-sg*Trp53*/Cre-R	TTGGCGCGCCGCGAATTCAAAAAAGCACCG
Lenti-sg*Trp53*/Cre-sgGirdin-F	AGCTTTGTTTAAACGAATTGACGCGTATTGGGAT
Lenti-sg*Trp53*/Cre-sgGirdin-R	GCCAAGCTCGGCGCGCCATT

**Table 2:** The sequence of primers for Lentivirus titer.

Primer	Sequences (5′ to 3′)
WPRE-F	TCAGCTCCTTTCCGGGACTT
WPRE-R	ATTGAGGGCCGAAGGGACGT
RagB-F	GCAAGACACCTTCATGGAAA
RagB-R	GCATGTCCTTTTCCAGTTCG

**Table 3:** The sequence of primers for genotyping.

Primer	Sequences (5′ to 3′)
*Kras*-F	CTAGCCACCATGGCTTGAGT
*Kras*-R	TCCGAATTCAGTGACTACAGATG
*LSL*-F	CGGCATGGACGAGCTGTACAAG
*LSL*-R	TCAGCAAACACAGTGCACACCAC
WT-F	CCCAAAGTCGCTCTGAGTTGTTA
WT-R	TCGGGTGAGCATGTCTTTAATCT

**Table 4:** The sequence of primers for siRNA and sgRNAs.

Primer	Sequences (5′ to 3′)
siGirdin-F	AAGAAGGCTTAGGCAGGAATT
siGirdin-R	AATTCCTGCCTAAGCCTTCTT
sgGiridn#1-F	CACCGGTCATGAACTGCTCCAGAA
sgGiridn#1-R	AAACTTCTGGAGCAGTTCATGACC
sgGiridn#2-F	CACCGCTAAAGCCACATACTCATCC
sgGiridn#2-R	AAACGGATGAGTATGTGGCTTTAGC

**Table 5:** Clinicopathological characteristics of LUAD patients.

Characteristic	Total	Girdin-Low (%)	Girdin-High (%)	*p* Value (*χ*^2^ Test)
**Number**	57	28 (49.1)	29 (50.9)	
**Age (y)**				
<55	27	12 (21.1)	15 (26.3)	0.5026
≥55	30	16 (28.1)	14 (24.5)	
**Gender**				
Male	32	13 (22.8)	19 (33.3)	0.1465
Female	25	15 (26.3)	10 (17.6)	
**Smoking**				
Yes	20	7 (12.3)	13 (22.8)	0.1168
No	37	21 (36.8)	16 (28.1)	
**Clinical stage**				
I	29	19 (33.3)	10 (17.6)	0.0118
II~IV	28	9 (15.8)	19 (33.3)	

## Data Availability

All data will be available upon reasonable request by emailing the corresponding author.

## Supplementary Materials

The following supporting information can be downloaded at: https://www.mdpi.com/article/10.3390/cancers14225688/s1. Figure S1 is the supplementary data for Figure 1; Figures S2–S5 are the uncropped WB figures for relevant results.
