# Supplementary material for: Girdin Promotes Tumorigenesis and Chemoresistance in Lung Adenocarcinoma by Interacting with PKM2
Source: Cancers (Basel). 2022 Nov 19;14(22):5688. doi: 10.3390/cancers14225688 (PMC9688487; doi:10.3390/cancers14225688)
Supplement: Supplementary file 1 [file cancers-14-05688-s001.zip › cancers-1867545-supplementary.pdf]

cancers

MDPI

Supplementary Material: Girdin Promotes Tumorigenesis and Chemoresistance in Lung Adenocarcinoma by Interacting with PKM2

Fuyang Cao, Desong Yang, Feiyu Tang, Can Lu, Xiang He, Songming Chen, Zhanghuan Yang, Siyuan Gong, Lunquan Sun, Atsushi Enomoto, Masahide Takahashi * and Liang Weng *

Supplementary Materials and Methods

Western blot

Cells were washed twice with cold PBS and treated with RIPA lysis buffer containing 25mM Tris-HCl (pH 7.4), 150 mM NaCl, 1% NP-40, 1% SDS and 0.5% sodium deoxycholate, supplemented with Complete Mini protease inhibitor and PhosSTOP phosphatase inhibitor cocktails (Roche, CA, USA). Lysates were collected by centrifugation at 12,000 g for 10 minutes at 4 °C, followed by adding 5x SDS sample buffer (50 mmol/L Tris-HCl, 10% SDS, 10 mmol/L EDTA, 0.1% bromophenol blue, and 30% glycerol; pH 6.8) and separated by SDS-PAGE. Proteins were transferred to PVDF membranes, blocked with 5% milk in PBS, incubated with primary antibodies, and detected by HRP-conjugated secondary antibodies (Dako, Carpinteria, CA, USA). All experiments were replicated at least three times, and we showed the representative data from the repeated experiments.

Immunoprecipitation

Cells were lysed with IP lysis buffer (20 mM Tris-HCl, 120 mM NaCl, 0.8% Triton X100, 1 mM EDTA, pH 7.4) containing Complete Mini Protease Inhibitor and PhosSTOP phosphatase inhibitor cocktails (Roche, California, USA). The lysates were clarified via centrifugation at 12,000g for 10 minutes and the supernatants were incubated with 2 μg primary antibody or normal IgG on a rotator at 4 ˚C overnight, followed by adding 20 μL protein A or G Sepharose beads (Sigma-Aldrich, St. Louis, USA) at 4 ˚C for 3 hours. Then the beads were washed with IP lysis buffer three times and eluted with 100 μL 1 × SDS sample buffer to collect protein complex.

Immunofluorescence Staining

A549 cells were plated on glass base dishes (Iwaki, Osaka, Japan), fixed, blocked, and incubated with Girdin and PKM2 antibodies at 4 ˚C overnight. The cells were washed with PBS for three times and then stained with Alexa Fluor 488 Goat Anti- Rabbit IgG and Alexa Fluor 594 Goat Anti-Mouse IgG (Invitrogen, California, USA) for 1 hour at room temperature. After incubation with DAPI to stain the nuclei, the cells were visualized with a confocal laser scanning microscope (LSM700, Carl Zeiss, Oberkochen, Germany).

Cell Proliferation Assay

Cell proliferation was monitored by counting viable cells with CCK-8 Cell Counting Kit (A311-01, Vazyme, Nanjing, China). Cells were seeded at 1000 cells per well in 96-well plates, CCK8 solution was diluted (1:10) in basic medium and added to the triplicate wells and then incubated at 37 °C for 1 hour. Subsequently, the absorbance at 450 nm was measured to calculate the number of vital cells in each well.

Colony Formation Assays

Cells were seeded into six-well plates at a concentration of 300 cells per well and cultured at 37 °C for 2 weeks. At the end of the incubation, the cells were fixed with 100%

methanol and stained with 0.1% crystal violet. Megascopic cell colonies were imaged and counted later.

Soft Agar Colony Formation Assay

1% and 0.6% noble agar (A5431, Sigma-Aldrich, St. Louis, USA) were prepared and sterilized before the experiment. 2x medium (1640: PM150110P, Procell, Wuhan, China; MEM: B9638, Sigma-Aldrich, St. Louis, USA) was combined with an equal volume of 1% low melting noble agar, in which FBS and penicillin-streptomycin-amphotericin B solution were contained. The mixture was spread 1.5 mL over each six-well plates and incubated at room temperature for 10 minutes.

Cells were counted and diluted into 2×10^4 cells/mL in complete medium, and then mixed rapidly and thoroughly with equal volume of 0.6% noble agar, 1.5 mL mixture was spread on the bottom agar. After solidification at room temperature, culture was continued for three weeks in a 37 °C incubator with 5% CO_2. Cell growth was observed, and colonies were imaged and counted.

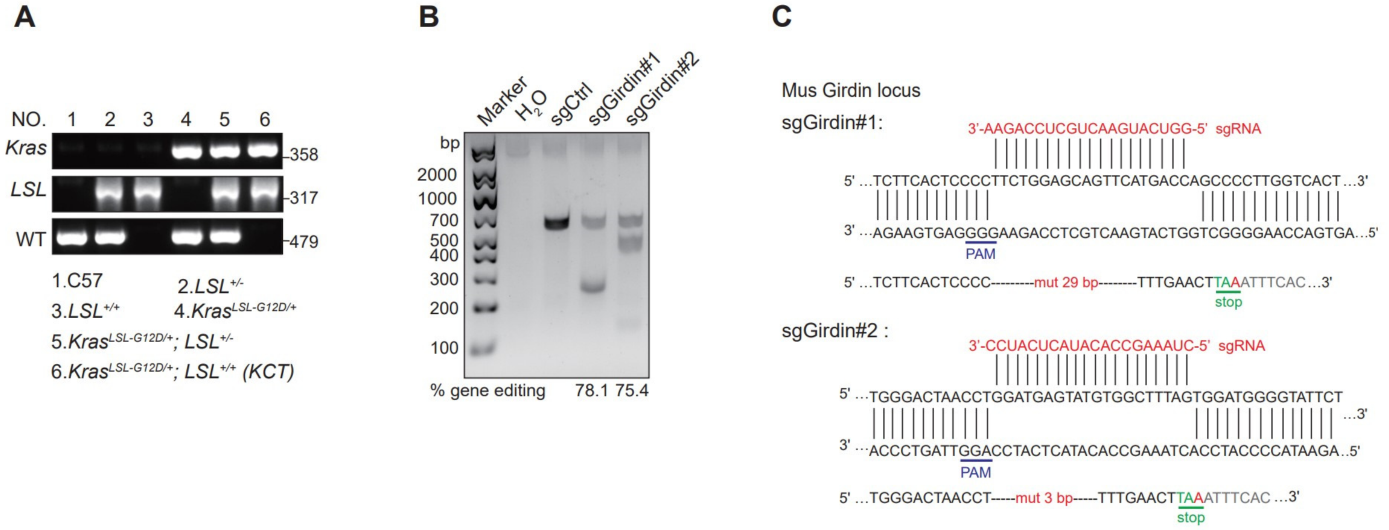

Figure S1. Girdin knockout significantly suppresses autochthonous tumorigenesis in LUAD. (**A**) Genotyping PCR of KCT mice (NO. 6). Heterozygosity in *Kras*$^{LSL\text{-}G12D/+}$ mice (NO.4, 5, 6); homozygosity in *Rosa26*$^{LSL\text{-}Cas9\text{-}tdTomato}$ mice (NO. 3, 6); heterozygosity in *Rosa26*$^{LSL\text{-}Cas9\text{-}tdTomato}$ mice (NO.2, 5), and WT (C57) allele (NO. 1, 4). (**B**) T7EI assay for Girdin sgRNAs in KP cells. The gene editing efficiency was calculated from the ratio of cleaved bands to the parental band (sgCtrl). (**C**) Schematic of CRISPR/Cas9 and representation of base-pairing between the sgRNAs and the targeting locus of Exon 1 and Exon 2 in the mouse *Girdin* gene. The sequence of the mutated alleles in sgGirdin#1 and sgGirdin#2 depicting the mutations (red and green) caused a nonsense mutation due to the early appearance of TAA (termination codon).

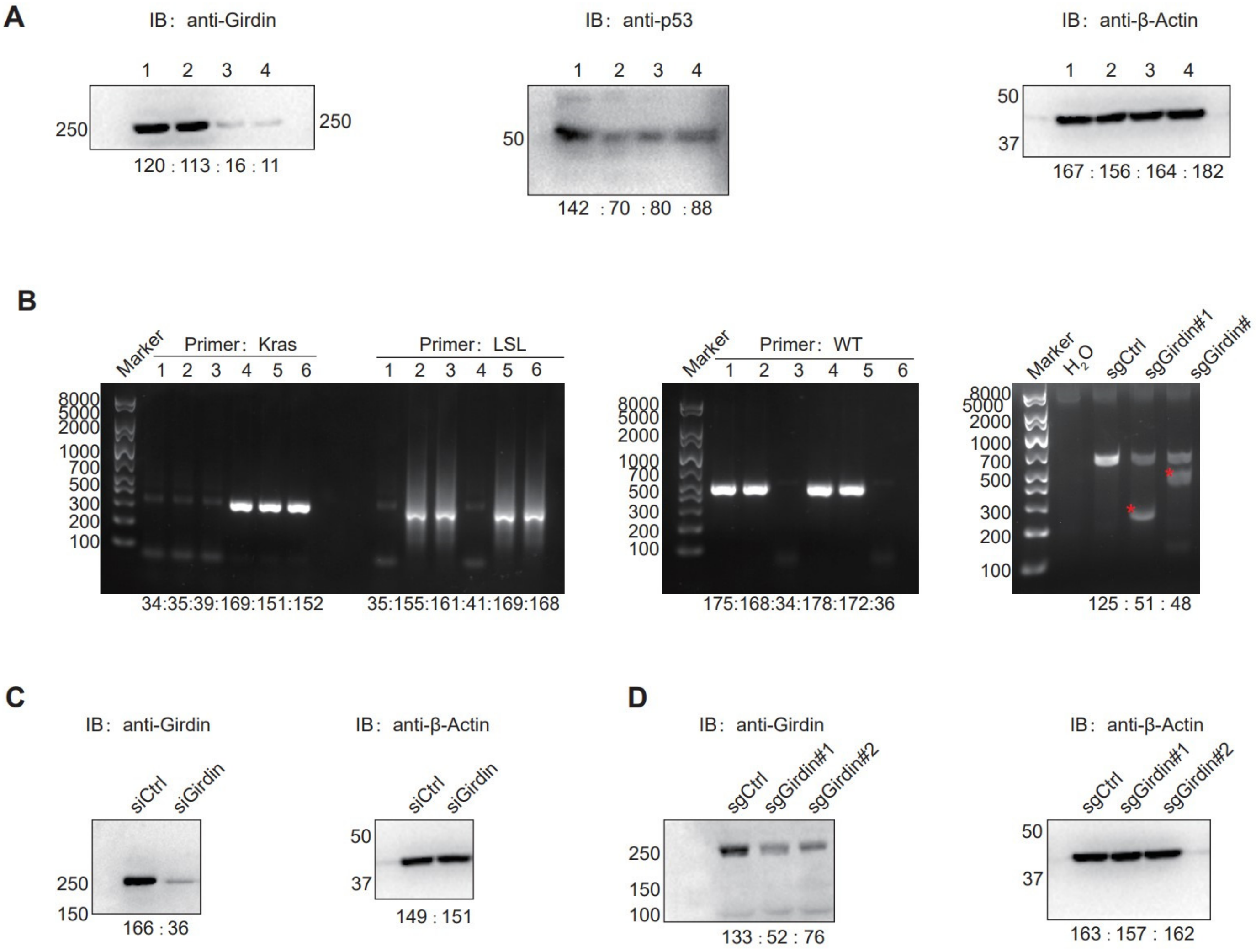

Figure S2. (**A**) The uncropped WB figures are for Figure 1C. (**B**) The uncropped genotyping figures are for Figure S1A and Figure S1B. Whole blots: (**C**) Figure 2F and (**D**) Figure 2L.

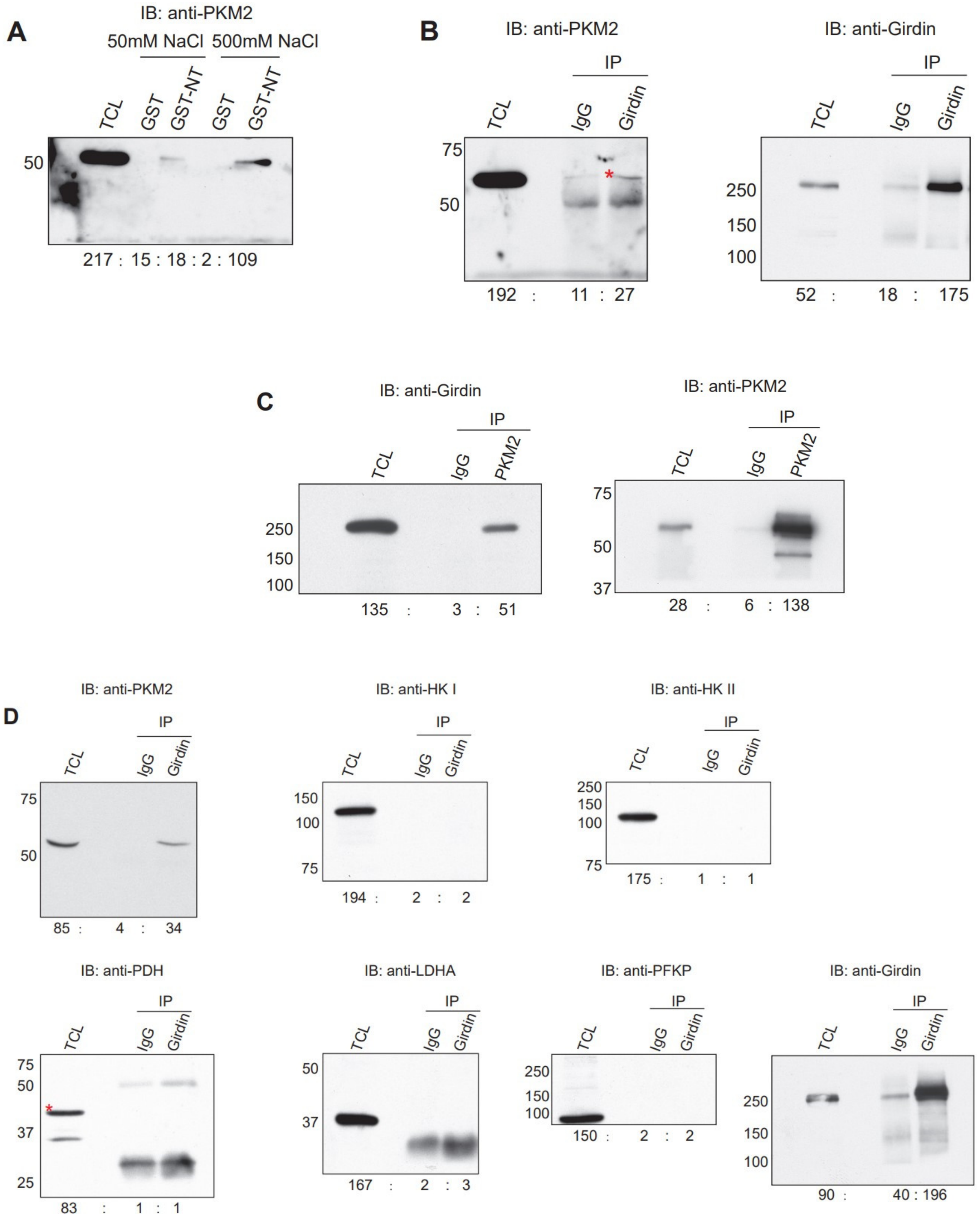
A
IB: anti-PKM2
50mM NaCl
500mM NaCl
TCL
GST
GST-NT
GST
GST-NT
50
217 : 15 : 18 : 2 : 109
B
IB: anti-PKM2
IP
TCL
IgG
Girdin
75
50
192 : 11 : 27
IB: anti-Girdin
IP
TCL
IgG
Girdin
250
150
100
52 : 18 : 175
C
IB: anti-Girdin
IP
TCL
IgG
PKM2
250
150
100
135 : 3 : 51
IB: anti-PKM2
IP
TCL
IgG
PKM2
75
50
37
28 : 6 : 138
D
IB: anti-PKM2
IP
TCL
IgG
Girdin
75
50
85 : 4 : 34
IB: anti-HK I
IP
TCL
IgG
Girdin
150
100
75
194 : 2 : 2
IB: anti-HK II
IP
TCL
IgG
Girdin
250
150
100
75
175 : 1 : 1
IB: anti-PDH
IP
TCL
IgG
Girdin
75
50
37
25
83 : 1 : 1
IB: anti-LDHA
IP
TCL
IgG
Girdin
50
37
167 : 2 : 3
IB: anti-PFKP
IP
TCL
IgG
Girdin
250
150
100
150 : 2 : 2
IB: anti-Girdin
IP
TCL
IgG
Girdin
250
150
100
90 : 40 : 196

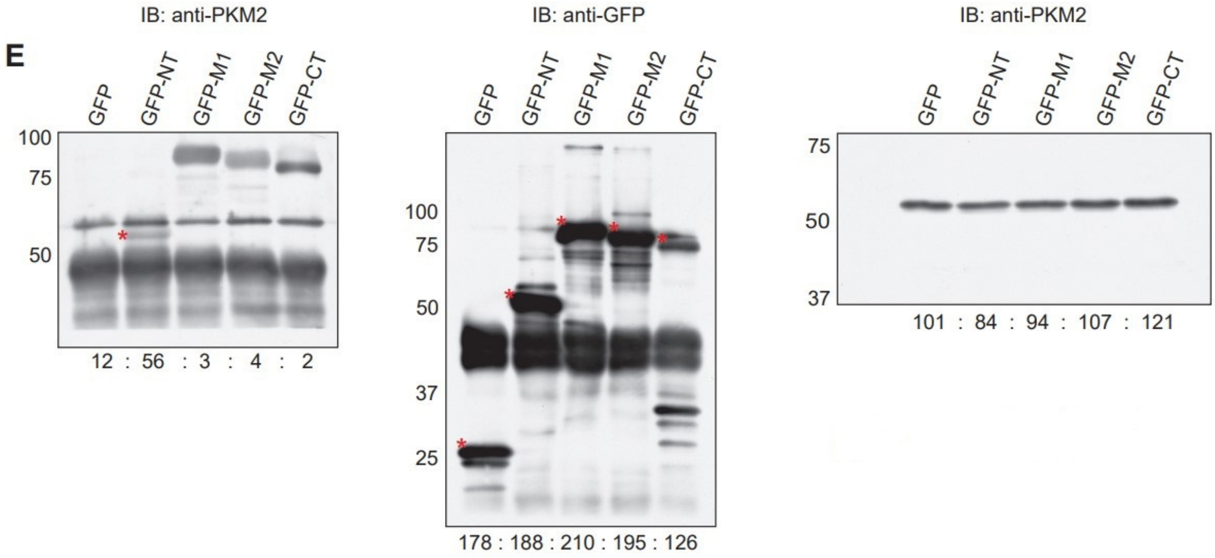

Figure S3. Whole blots: (**A**) Figure 4B, (**B**) Figure 4C, (**C**) Figure 4D, (**D**) Figure 4E and (**E**) Figure 4H.

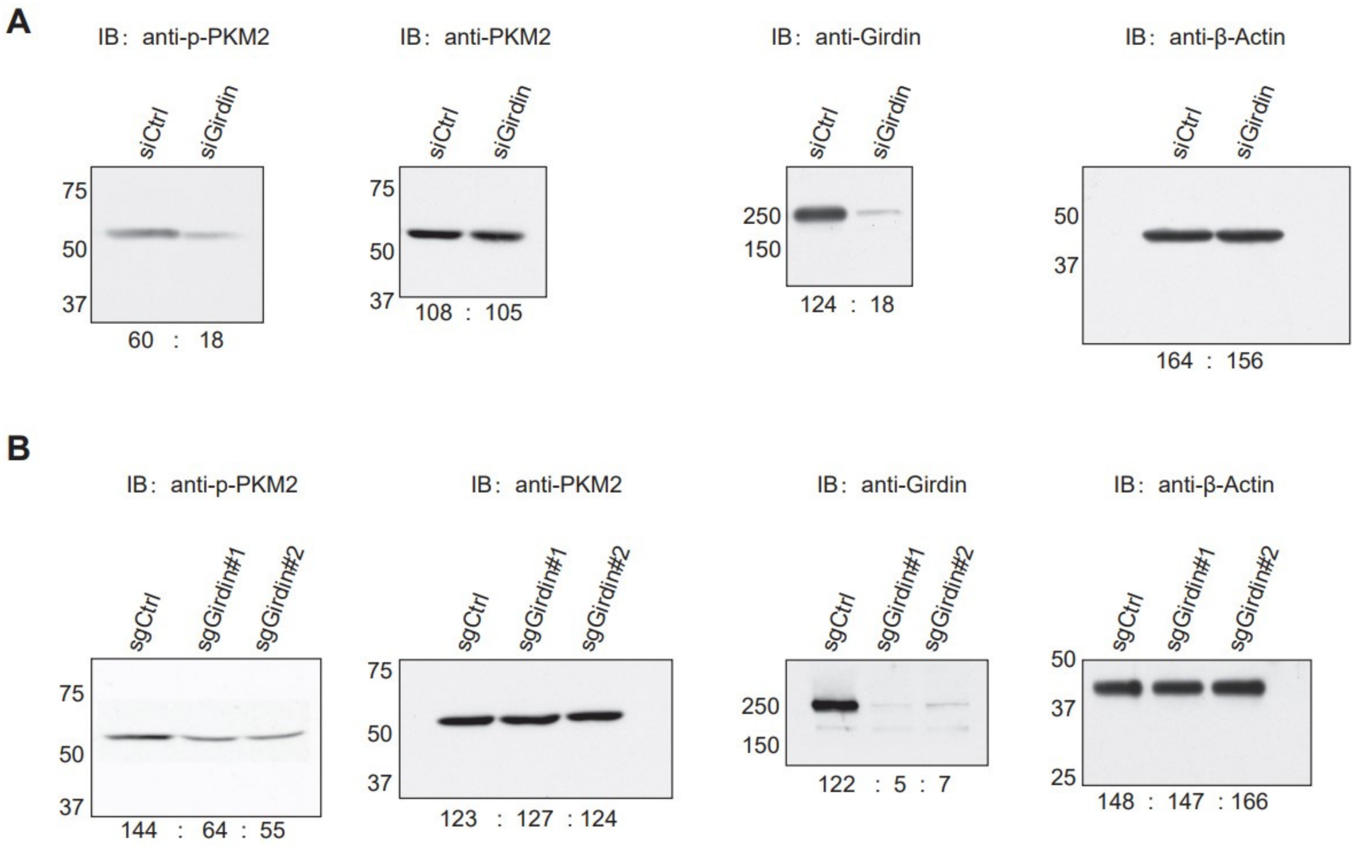

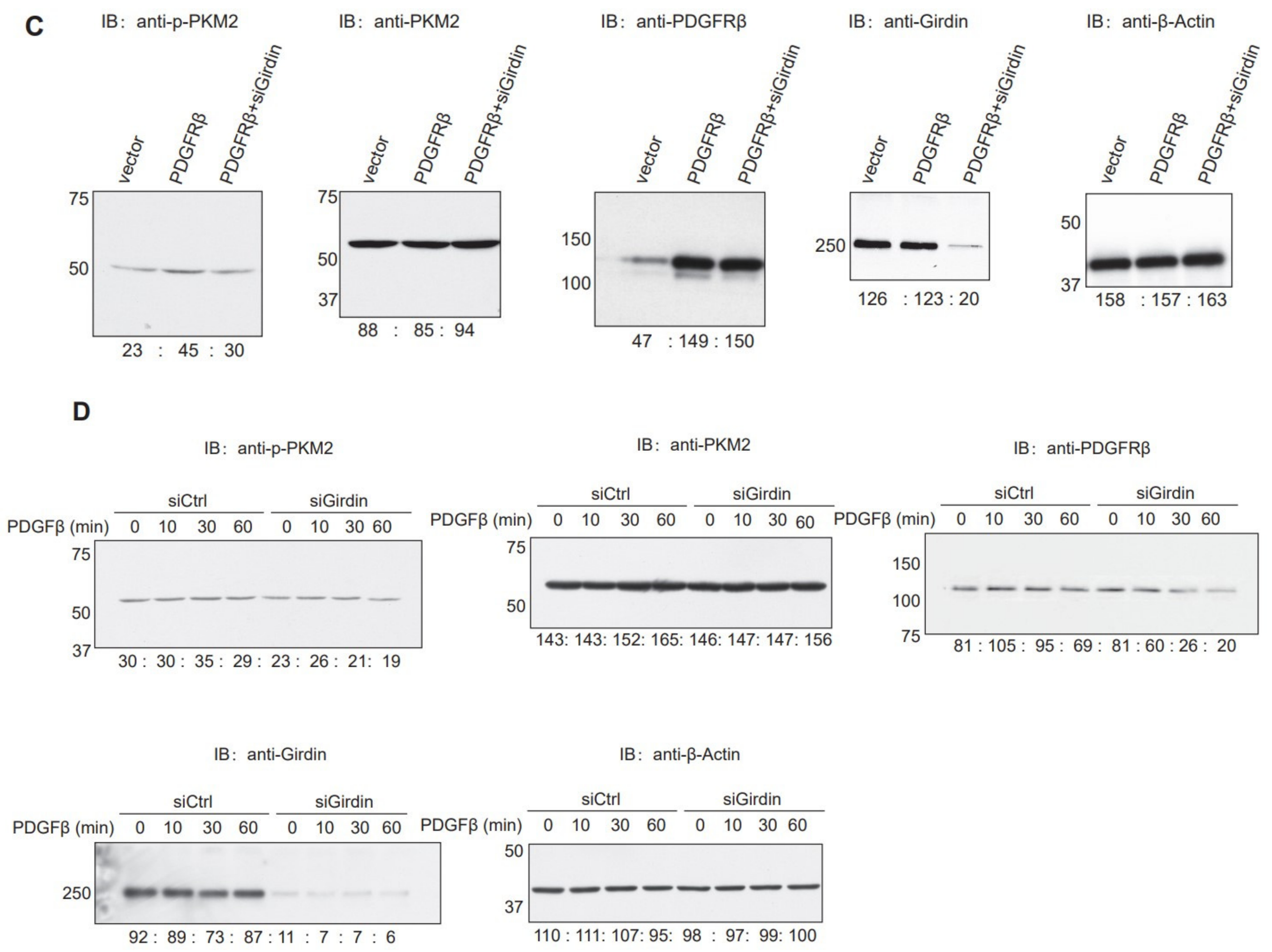

Figure S4. The uncropped WB figures are as follows: (**A**) Figure 5A, (**B**) Figure 5B, (**C**) Figure 5E and (**D**) Figure 5F.

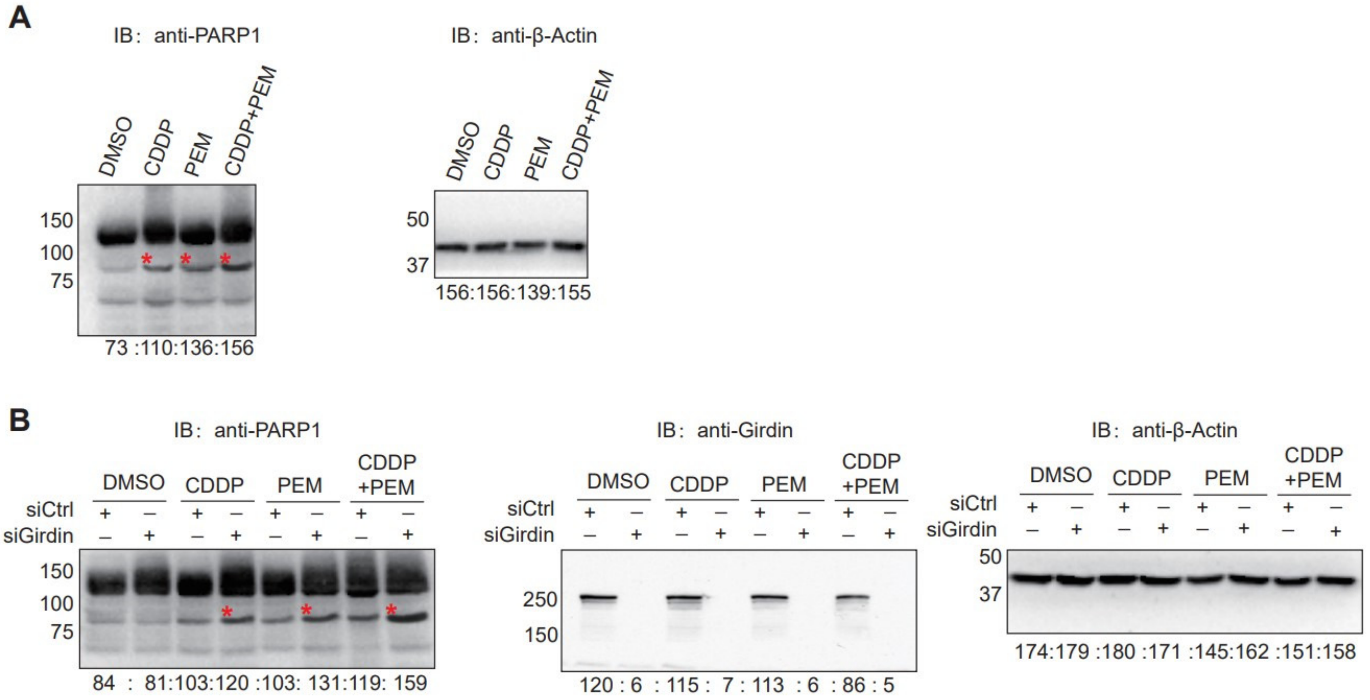

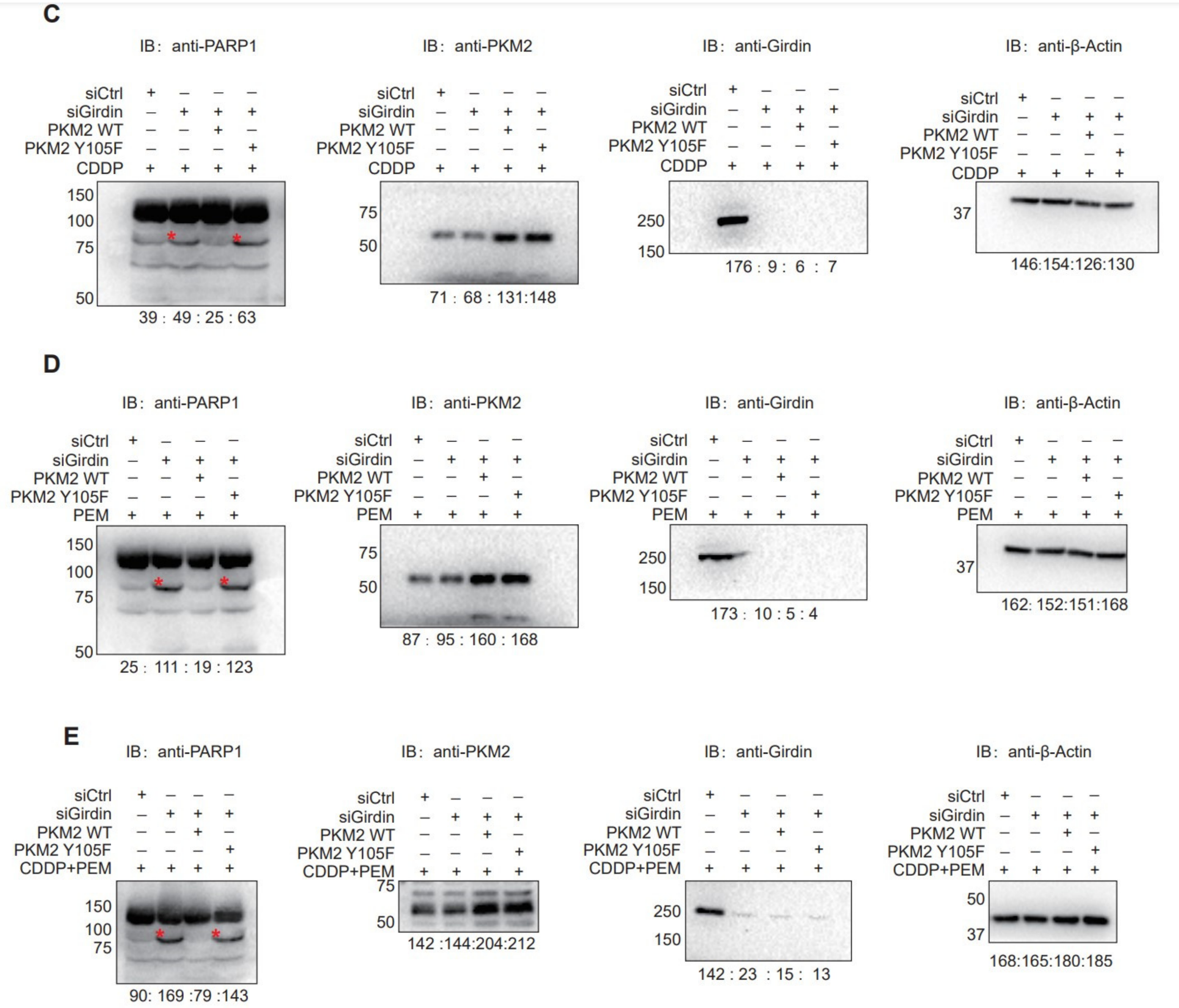

Figure S5. The uncropped WB figures are as follows: (**A**) Figure 5H, (**B**) Figure 5I, (**C**) Figure 5J, (**D**) Figure 5K and (**E**) Figure 5L.
